# Prenatal Adversities and Latino Children’s Autonomic Nervous System Reactivity Trajectories from 6 Months to 5 Years of Age

**DOI:** 10.1371/journal.pone.0086283

**Published:** 2014-01-21

**Authors:** Abbey Alkon, W. Thomas Boyce, Linh Tran, Kim G. Harley, John Neuhaus, Brenda Eskenazi

**Affiliations:** 1 School of Nursing, University of California San Francisco, San Francisco, California, United States of America; 2 School of Population and Public Health, Faculty of Medicine, University of British Columbia, Vancouver, Canada; 3 University of California, Berkeley, School of Public Health, Berkeley, California, United States of America; 4 Center for Environmental Research and Children’s Health (CERCH), Berkeley, California, United States of America; 5 UCSF Epidemiology and Biostatistics, University of California San Francisco, San Francisco, California, United States of America; 6 CERCH, School of Public Health, University of California, Berkeley, California, United States of America; Hunter College, City University of New York (CUNY), CUNY School of Public Health, United States of America

## Abstract

The purpose of the study was to determine whether mothers’ adversities experienced during early pregnancy are associated with offspring’s autonomic nervous system (ANS) reactivity trajectories from 6 months to 5 years of age. This cohort study of primarily Latino families included maternal interviews at 13–14 weeks gestation about their experience of a range of adversities: father’s absence, general social support, poverty level, and household density. ANS measures of heart rate, respiratory sinus arrhythmia (parasympathetic nervous system) and preejection period (sympathetic nervous system) were collected during resting and challenging conditions on children at 6 months and 1, 3.5 and 5 years of age. Reactivity measures were calculated as the mean of the responses to challenging conditions minus a resting condition. Fixed effects models were conducted for the 212 children with two or more timepoints of ANS measures. Interactions between maternal prenatal adversity levels and child age at time of ANS protocol were included in the models, allowing the calculation of separate trajectories or slopes for each level of adversity. Results showed no significant relations between mothers’ prenatal socioeconomic or social support adversity and offspring’s parasympathetic nervous system trajectories, but there was a statistically significant relationship between social support adversity and offspring’s heart rate trajectories (p<.05) and a borderline significant relationship between socioeconomic adversity and offspring’s sympathetic nervous system trajectories (p = .05). Children whose mothers experienced one, not two, social support adversity had the smallest increases in heart rate reactivity compared to children whose mothers experienced no adversity. The children whose mothers experienced no social support and no socioeconomic adversity had the largest increases in heart rate and preejection period respectively from 6 months to 5 years showing the most plasticity. Mothers’ prenatal adverse experiences may program their children’s physiologic trajectory to dampen their heart rate or sympathetic responsivity to challenging conditions.

## Introduction

Exposure to adverse conditions during the prenatal period can alter the neurochemistry of the fetus’ central nervous system in ways that affect emotional and cognitive regulation, motor development and stress responsiveness [1]–[4]. Adversity experienced during sensitive periods of fetal development can program a fetus’ neurological development and affect their life course, resulting in an increased risk of disease later in life [5]. Fetal programming has been defined as a “… process by which a stimulus or insult during a vulnerable developmental period has a long-lasting or permanent effect” [6]. Studies of ‘fetal programming’ [5], [6] primarily focus on the effects of adversity on children’s cortisol, an end product of hypothalmic-pituitary-adrenal (HPA) stimulation. Cortisol crosses the blood-brain barrier and thus, it has direct effects on the brain. Mothers with high cortisol during pregnancy have children with high cortisol reactivity at 2 days and 10 months of age [7] and at five years of age [8]. In animal studies, the stress related to overcrowding during pregnancy was associated with altered glucocorticoid functioning (i.e. increased cortisol) [9]. Although there are several studies of children’s cortisol responses to prenatal adversity [6], [7], [10], [11], there are few studies of children’s autonomic nervous system (ANS) responses to examine broad neurobiologic responses to prenatal adversity [12], [13].

The ANS is comprised of the parasympathetic and sympathetic nervous systems. The parasympathetic nervous system provides a restorative function and helps the body maintain a steady state while the sympathetic nervous system provides the ‘fight or flight’ response and is activated under conditions of stress [14]. When a mother experiences adversity during pregnancy, she may be programming her fetus to detect threats and adjust its ANS developmental trajectory for survival [15]. Studies of young children’s ANS responses may improve our understanding of these complex, life-altering processes.

Studies of prenatal adversity in animals and humans show changes in offspring’s ANS and motor development. In an animal study, prenatal physical stressors increased offspring’s blood pressure under stressful conditions and recovery [16]. In a primate study where stress during early pregnancy was compared to late pregnancy, early adversity predicted more pervasive impairments of offspring’s motor development [17]. These results were supported by a study in humans where adversity during early pregnancy predicted accelerated maturation of the infant’s motor development and activation of the parasympathetic nervous system (i.e. increased heart rate variability) [4]. On the other hand, one study of cumulative prenatal adversity at 16 weeks gestation was not associated with children’s resting parasympathetic and sympathetic nervous system using measures of respiratory sinus arrhythmia (RSA) or preejection period (PEP) respectively at 5 years of age [13].

It is postulated that early life experiences, such as those resulting from social support and economic adversity [18], [19] can get “under the skin” and change children’s biology during sensitive developmental periods [20] when the developing brain is more receptive to a variety of environmental signals, both positive and negative [1]. Social adversities have been associated with children’s parasympathetic withdrawal and increased HR [21] while socioeconomic adversities have been associated with muted cardiovascular reactivity [19]. Infants and young children have immature stress- and response- systems which exhibit plasticity, such that these systems change over time in response to their environments. Moreover, adversities experienced early in life might increase the sensitivity of children’s developing threat appraisal system, thus, enhance children’s vulnerability to stressors throughout life and decrease plasticity [22], [23].

A few studies show differences in ANS responses for African American versus European American school-age children [24]–[26], but there are no studies on Latino children’s ANS responses. Since Hispanics and Latinos comprise more than 25% of the children under five years of age in the United States (U.S.) [27], it is particularly important to include Latino children in studies of adversity and ANS responsivity [21].

The ANS is developing and changing during the early years of life [28], yet it is not known if early experiences of prenatal adversity enhance or dampen children’s developmental trajectory of the ANS. Children’s biologic sensitivity to their environment, as measured by cardiac indices of the ANS during resting and challenging conditions, is driven by their conditional adaptation to prenatal and postnatal impoverished adverse environments [4], [29]–[32]. Therefore, the present study contributes to this broader literature and also to the limited body of research on Latino mothers’ adversities experienced during early pregnancy and their children’s ANS reactivity trajectories. The study hypotheses are: (1) mothers who experienced high social support adversity during their early pregnancy will have young children who have less plasticity of their ANS (i.e. heart rate (HR), respiratory sinus arrhythmia (RSA) and preejection period (PEP)) reactivity trajectories (i.e. small slope/change) in the first five years of life than children whose mothers experienced no social support adversity and (2) mothers who experienced high socioeconomic adversity during early pregnancy will have young children who have less plasticity of their ANS reactivity trajectory in the first five years than children whose mothers experienced no adversity.

## Materials and Methods

### Sample

This Center for the Health Assessment of Mothers and Children of Salinas (CHAMACOS) study is a longitudinal birth cohort study of the effects of environmental exposures on the health of pregnant women and their children living in an agricultural community in California [33]. Pregnant women were recruited between October 1999 and October 2000 through six prenatal clinics that serve a predominantly low-income, Spanish-speaking population. Eligible women were 18 years of age or older, less than 20 weeks gestation, Spanish or English speaking, Medi-Cal eligible, and planning to deliver at the county hospital.

Of the 601 women initially enrolled, 526 were followed through delivery with 537 live births. Four hundred and twenty eight of their infants were seen at the 6-month old visit, 418 at 1 year, 330 at 3.5 years, and 319 at 5 years of age. The University of California, Berkeley’s and San Francisco’s Centers for Protection of Human Subjects approved the study procedures, including consent procedures and forms. Consent forms were read and reviewed with the mothers before each phase of data collection and then the mothers signed the consent form. The larger study’s hypothesis that exposure to organophosphate pesticides (OP) would affect children’s ANS reactivity was assessed by measuring urinary dialkylphosphate metabolites in the children’s urine at the same time the ANS protocol was administered. Cross-sectional associations between OP and ANS were analyzed using multiple regression models and cumulative OP exposures were assessed using area-under-the-concentration-time-curve (AUC) methodology and longitudinal mixed models. The relation between exposure to OP and children’s ANS was shown to not be significant [34], therefore exposure is not controlled for in these analyses.

### Prenatal Adversities

At study enrollment during 13–14 weeks gestation, mothers completed a baseline interview with a trained psychometrician in either English or Spanish. The interview included demographic, social, family, and economic measures [33], [35] identified in the literature as adversities that impact a child’s physiologic, physical or emotional development [4], [29], [36]–[38]. The social support index of adversity consisted of two measures: level of social support perceived by the mother and father’s presence (or absence) during the pregnancy. The mother’s level of social support was assessed by 8 items from the Duke-University of North Carolina Functional Social Support Questionnaire (FSSQ) [39], with items such as “I have people who care what happens to me”. Mother’s identified each item on a Likert scale from 5 (as much as I would like) to 1 (much less than I would like). Since the FSSQ includes dimensions of emotional social support, two additional items of instrumental support were added (i.e. “I have help around the house or with child care; I have people who help me when I can’t make ends meet.”). The final 10-item, modified version of the FSSQ had good internal consistency (Cronbach’s α = 0.86) [35]. The mean of the modified FSSQ were dichotomized above and below 3.2 (range 1.2 to 5) based on the distribution of less than <30% as low support (Table 1). Father’s presence during the pregnancy was assessed when the mother was asked, “Since you became pregnant, have you lived with the baby’s father …?” The mothers’ responses were dichotomized as present (i.e. some, most or all of the time) or not present (i.e. not at all) (Table 1).

**Table 1:** Frequency of Social Support and Socioeconomic Adversity Variables and Indices (N = 212).

Social Support Variables and Index	N (%)
**Mother Living with Baby’s Father During Pregnancy**	
Not Present/Absent	53 (25%)
Some, Most or All of the Time	159 (75%)
**Overall Social Support Score:** modified FSSQ	
<3.2 mean	62 (29%)
> = 3.2 mean	150 (71%)
Social Support Index*: modified FSSQ, Father not present/absent during pregnancy	
No adversity	121 (57%)
1 Adversity	74 (35%)
2 Adversities	17 (8%)
**Socioeconomic Variables and Index**	N (%)
**Poverty Level**	
At or below poverty level	132 (62%)
Above poverty level	80 (38%)
**Household Density**	
More than 1.5 persons per room	91 (43%)
Less than or equal to 1.5 persons per room	121 (57%)
**Socioeconomic Status Index:** poverty level, household density	
No adversity	55 (26%)
1 Adversity	100 (47%)
2 Adversities	57 (27%)

The socioeconomic index of adversity consisted of measures of poverty (i.e. living at or below the poverty level versus above the poverty level) and household density (i.e. number of people living in the household divided by the number of rooms, excluding bathrooms) (Table 1). Poverty level was calculated by dividing household income by the number of people supported by that income and comparing that number to the federal poverty levels (FPL) [40].

Each adversity index (socioeconomic, social support) included two variables dichotomized, equally weighted, and summed together, yielding a range from 0 to 2 (Table 1). High social support adversity referred to low scores on the modified FSSQ and/or fathers’ absence during the pregnancy. High socioeconomic adversity included mothers who lived at or below 100% FPL and/or in crowded households with ≥1.5 persons per room.

### ANS Measures

The ANS protocol was completed for about 50% of the enrolled children at 6 months (n = 161), 1 year (n = 155), and 3.5 years (n = 136) due to limited funds (for more details, see article) [28]. When our funds increased, we were able to include all the children during the 5 year visit (n = 297). This resulted in ANS measures at one timepoint for 378 children and two or more timepoints for 212 children.

The ANS protocols included measures of heart rate (HR), respiratory sinus arrhythmia (RSA) (parasympathetic nervous system) and preejection period (PEP) (sympathetic nervous system) during resting and challenging conditions modified from standardized, valid and reliable protocols [41], [42]. The social, cognitive, physical, and emotional challenges represented normative, common stressors for each developmental period. At 6 months and 1 year of age, the 7-minute protocol included three challenges (jack-in-the-box, vibrator on the leg, listening to the audio of a sick baby crying) preceded and followed by resting states, listening to a lullaby [42]. At 3.5 and 5 years of age, the 15-minute protocol included four challenges (social interview, number recall, concentrated juice applied to tongue, emotion-evoking video) preceded and followed by resting states, stories read aloud [41].

The protocols were administered by bilingual, bicultural staff in private rooms located in a research office next to the local hospital or a Recreational Vehicle redesigned as a research lab parked at the participant’s home. The protocol was administered at the 6- and 12-month timepoints in the language spoken at home, either Spanish or English. At the 42- and 60-month timepoints the protocol was administered in the child’s language of choice. Inter-rater reliability on the administration of the protocol was assessed four times each year through on-site observations or videotape reviews by two research staff.

Band electrodes were used to collect the ANS data at 6 months and 1 year [42] and spot electrodes at 3.5 and 5 years [41]. The tetrapolar configuration of electrodes included placement of two bands on the neck and two bands on the trunk to collect impedance, electrocardiograph (ECG) and respiratory measures at 6 months and 1 year. At 3.5 and 5 years, two spot electrodes were placed 3 cm apart on the neck and trunk to collect impedance and respiratory measures, and spot electrodes were placed on the right clavicle and lower left rib for ECG measures.

Data were acquired at 6 months and 1 year of age using the Minnesota Impedance Cardiograph HIC-2000 and at 3.5 and 5 years of age using the Biopac MP150, since the HIC-2000 was no longer available. Continuous measures of HR, ECG, Z_o_ (basal impedance) and d*Z*/d*t* (first derivative of the impedance signal) were collected during the protocol. A 4- milliamp AC current at 100Hz was passed through the two outer, current electrode bands/spots and Z_o_ and dZ/d*t* signals were acquired from the two inner voltage-recording bands/spots. The data were filtered, extracted, and then scored using the ANS suite software at 6 months and 1 year [43] and Mindware (www.mindware.org) at 3.5 and 5 years of age.

HR was measured as beats per minute. HR is an integrated measure sensitive to both the parasympathetic and sympathetic branches of the ANS.

RSA is the periodic oscillation in sinus rhythm occurring at the frequency of respiration and manifested as an increase in HR with inspiration and a decrease during expiration. As the parasympathetic influence on HR decreases, referred to as parasympathetic withdrawal, the RSA index decreases. RSA scores were calculated using the interbeat intervals on the ECG reading, respiratory rates derived from the impedance (e.g. d*Z*/d*t*) signal, and a bandwidth range of 0.24 to 1.04 Hz at 6 months and 1 year and 0.15 to 0.80 Hz at 3.5 and 5 years of age [44].

PEP is an indirect, noninvasive cardiac measure of the sympathetic nervous system’s influence on the cardiac cycle. PEP is the time interval, measured in milliseconds, of the duration of isovolumetric contraction in the left ventricle and it represents the sympathetic influence on the contractility of the heart. As sympathetic activity increases, PEP decreases. PEP is the time interval between the Q point on the ECG wave and the B point on the d*Z*/d*t* wave.

Data cleaning procedures included checking all outliers (>3SD) minute-by-minute and summary scores and deleting a child’s data at that timepoint if more than 25% of their minutes were not scored. The percentage of children with clean, scored ANS data varied by age: 6 months (83%), 1 year (86%), 3.5 years (93%), 5 years (95%). Missing data were due to child or parent refusals, equipment failure, or noisy data due to child movement or electrode displacement. Six children with the medical diagnosis of seizures were dropped. The cohort included four sets of twins. ANS data were available on 161 at 6-month olds, 155 at 1 year, 136 at 3.5 years, and 297 at 5 years of age.

Reactivity or difference scores were calculated as the mean response across the challenges minus the mean of the first resting state [45]. Reactivity trajectories were calculated by estimating the mean (SD) slope and intercept for each child across the ages [28].

### Data Analysis

Descriptive statistics were calculated for all demographic characteristics.

Conditional likelihood methods/fixed effects models [46] were used to analyze the relationship between levels of prenatal adversities and HR, RSA, PEP reactivity trajectories (i.e. slope) from 6 months to 5 years of age for each child. Interactions between maternal prenatal adversity levels and time (i.e. child age at time of the ANS protocol) were included in the models, allowing the calculation of separate slopes for each level of adversity. Because conditional likelihood methods rely only on the variation of predictors and outcomes within each child, children with only one observation were excluded from the analyses. Therefore, the conditional likelihood methods included 212 children who completed the ANS protocol at 2 or more timepoints, including 68 with 3 ANS timepoints and 43 children with 4 ANS timepoints. Seventy-five percent of the children in the analyses had ANS data collected at both infancy (6 months and 1 year) and the preschool ages (3.5 and 5 years). Lastly, secondary fixed effect models were conducted to determine if the slopes of the ANS trajectories differed from zero in the significant models.

The conditional likelihood methods were preferred over the more commonly used mixed effects (i.e. fixed and random effects) models since conditional likelihood methods did not require positive correlations of the ANS measures (i.e. HR, RSA, PEP) within individuals across time. In this study, the ANS measures were not consistently positively correlated across time. Also, this study’s objectives did not necessitate estimating and/or analyzing between-person effects. The conditional likelihood methods estimated the effect of prenatal adversity on children’s ANS changes over time while simultaneously controlling for time-invariant covariates (e.g. sex) and adjusting for the lack of independence among the multiple observations for each child. We fit the conditional likelihood methods and estimated model parameters using maximum likelihood methods and routines in Stata 11.

The reasons for missing ANS values are consistent with the assumption that the data were missing completely at random. The majority of missing ANS values were missing by the sampling design. Maximum likelihood procedures were employed to provide accurate estimates of missing data [47].

Since the conditional likelihood analyses assessed ANS trajectories but could not control for changes in adversity over time, post-hoc analyses were conducted to assess the stability of adversity over time and the cross-sectional relations between adversities and ANS reactivity at 6 months, 1, 3.5 and 5 years of age. The stability of the prenatal adversities measured at 6 months, 1, 3.5, and 5 years of age were analyzed using Spearman correlations. The cross-sectional relations between adversities and ANS reactivity at 6 months, 1, 3.5 and 5 years of age were analyzed using multiple regression models.

## Results

### Child and Family Characteristics

The majority of the children (N = 378) were male (51%), Latino (97%), fullterm (93%) and not low birthweight (96%). The majority of the mothers had one other living child (68%), less than a high school education (80%), spoke mostly Spanish at home (90%), were under 30 years of age (75%), and lived in the U.S. less than 5 years (53%). There were no significant demographic differences between the 212 children included in these analyses and the full CHAMCOS sample (n = 537). HR, RSA and PEP reactivity trajectories (i.e. slopes) did not significantly differ by the children’s sex. Thus, subsequent models did not include children’s sex.

### ANS Trajectories by Prenatal Social Support Adversity

**Figure 1:**
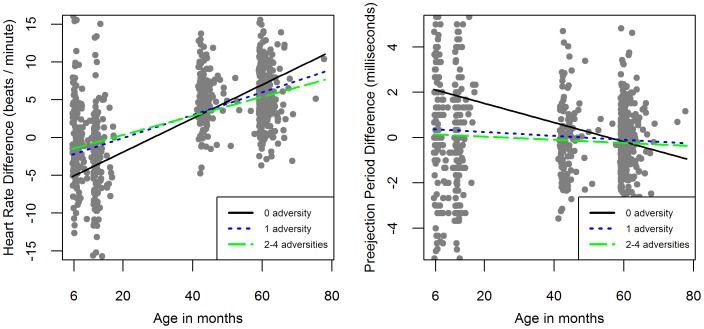
Social Support and Socioeconomic Adversity by HR and PEP Reactivity Trajectories.

Although there were no significant relations between social support adversity and RSA and PEP reactivity trajectories, there were significant relations between mothers’ prenatal level of social support adversity and children’s HR reactivity trajectories (Figure 1; Table 2). The children living with mothers who experienced one social support adversity had significantly different HR reactivity trajectories than children living with mothers who experienced no social support adversity (beta coefficient (SE) = −0.69 (0.28), p<.05) (Table 2). Overall, children living with mothers who experienced high social support adversity (i.e. low modified FSSQ scores, fathers’ absence) during their pregnancy (index score = 1,2) had higher HR reactivity at 6 months of age than children whose mothers had no social support adversity but by 5 years of age these children had lower HR reactivity than children whose mothers had no social support adversity (Figure 1). Although the children in all 3 social support adversity groups (i.e. 0, 1, 2) had significant positive slopes in their HR reactivity from 6 months to 5 years of age (Figure 1), the children whose mothers experienced no social support adversity (i.e. 0) showed the steepest HR reactivity slopes from 6 months to 5 years of age (beta coefficient (SE) = 2.06 (0.17), p<.05) (Table 3).

**Table 2:** Conditional likelihood estimates of the children’s ANS trajectories from 6 months to 5 years of age by prenatal socioeconomic and social support adversity**.**

Independent Variable:Prenatal Adversity	Dependent Variable:ANS Trajectory Conditional Likelihood Slope (beta coefficient (SE))		
Social Support	HR Reactivity (n = 211)	RSA Reactivity(n = 211)	PEP Reactivity (n = 202)
One vs. No adversity	−0.69 (0.28)^a^	−0.00 (0.03)	0.06 (0.16)
Two vs. No adversity	−0.58 (0.40)	0.03 (0.04)	0.09 (0.23)
Two vs. One adversity	0.10 (0.43)	0.03 (0.05)	0.03 (0.24)
**Socioeconomic status**	**HR Reactivity (n = 211)**	**RSA Reactivity** **(n = 211)**	**PEP Reactivity (n = 202)**
One vs. No adversity	−0.48 (0.33)	0.04 (0.04)	0.37 (0.19)^b^
Two vs. No adversity	−0.56 (0.35)	0.05 (0.04)	0.30 (0.20)
Two vs. One adversity	−0.08 (0.29)	0.02 (0.03)	−0.07 (0.16)

a Social support predicts HR reactivity trajectory p<.05, Overall model: F(3,358) = 69.13, p<.05, n = 211.

b SES index predicts PEP reactivity trajectory p = .05, Overall model: F(3,340) = 2.78, p<.05, n = 202.

**Table 3:** Conditional likelihood estimates of the children’s HR and PEP trajectories from 6 months to 5 years of age by prenatal socioeconomic and social support adversity: Comparison of HR and PEP trajectory slopes (beta coefficient) to zero slopes.

Independent Variable	Dependent Variable: ANS Trajectory Conditional Likelihood Slope (beta coefficient (SE))	
Level of PrenatalAdversity	Social Support Adversity predictingHR Reactivity Trajectories (n = 211)	Socioeconomic Adversity predictingPEP Reactivity Trajectories (n = 202)
No adversity	2.06 (0.17)^a^	−0.42 (0.16)^a^
One adversity	1.37 (0.23)^a^	−0.05 (0.10)
Two adversities	1.48 (0.36)^a^	−0.12 (0.12)

a p<.05 p-value indicates whether the slope was significantly different than zero (no slope).

### ANS Trajectories by Prenatal Socioeconomic Adversity

There were no significant relations between socioeconomic adversity and HR reactivity and RSA reactivity trajectories. There was a borderline significant relation between prenatal mothers’ level of socioeconomic adversity and children’s PEP reactivity trajectories (Figure 1; Table 2).

Children whose mothers experienced any level of prenatal socioeconomic adversity demonstrated no significant change in their PEP reactivity from 6 months to 5 years of age. However, children whose mothers did *not* experience prenatal socioeconomic adversity demonstrated a significant negative slope in PEP reactivity across time, changing from being the least reactive at 6 months of age to the most reactive at 5 years of age (beta coefficient (SE) = −0.42 (0.16), p<.01) (Figure 1; Table 3). Larger negative PEP reactivity scores reflects greater sympathetic activation during challenging conditions compared to their resting states. There was also a borderline significant difference in the no adversity versus one adversity group’s slopes (beta coefficient (SE) = 0.37(0.19), p = .05) (Table 2).

The post-hoc stability of the prenatal adversities across the four postnatal timepoints (i.e. 6 months and 1, 3.5, 5 years) was assessed with correlations between each age. They showed that fathers’ absence was moderately correlated (rho = .4) from the prenatal to the postnatal timepoints but was stronger between the postnatal timepoints (rho range = .5 to.7). Mothers’ social support from prenatal to postnatal (at 1 and 5 years) was moderately correlated (rho range = .4 to.5). Household density from prenatal to postnatal was weakly to moderately correlated across the postnatal period (rho range = .2 to.5). Poverty level from prenatal to postnatal was weakly to moderately correlated across the postnatal period (rho range = .2 to.4).

In the cross-sectional analyses, there were no significant relations between socioeconomic and social support prenatal adversities and HR, RSA or PEP reactivity.

## Discussion

Children’s ANS reactivity trajectories between 6 months and 5 years of age differed by their mothers’ prenatal exposure to social support and socioeconomic adversities. The findings supported the hypothesis that mothers who experienced high social support adversity during their early pregnancy had young children with less plasticity of their HR reactivity trajectories (i.e. small slope/change) in the first five years of life than children whose mothers experienced no social support adversity. The hypothesis was not supported for the relations between mother’s social support adversity and children’s PEP or RSA reactivity trajectories. Additionally, the findings supported the hypothesis that mothers who experienced high socioeconomic adversity during early pregnancy had young children who have less plasticity of their sympathetic nervous system (i.e. PEP) reactivity trajectory in the first five years than children whose mothers experienced no adversity. This hypothesis was not supported for children’s HR or RSA reactivity. These findings show that the mothers who experienced no prenatal adversity had children who developed a heightened sensitivity to challenging conditions as they grew older, while the mothers who experienced prenatal adversities had children with less reactivity and a dampened physiologic response under challenging conditions (i.e. smaller slopes from 6 months to 5 years of age).

Our findings support prenatal programming as a potential process by which prenatal adversity experienced during pregnancy, a vulnerable developmental period, affects offspring’s physiology which ultimately changes a child’s future health and behavior [6], [48]. In other studies with related findings, children living with mothers who experienced perinatal adversities showed conflicting findings. Some studies showed the children experienced physiologic hyper-responses and other studies showed no response or hypo-responses. Some studies of children exposed to prenatal stressors had high cortisol reactivity during infancy [49], [50] and adolescence [10]. In other studies, children exposed to prenatal adversities showed a dampened response of their HPA axis during the neonatal period [51] and attenuated blood pressure reactivity during adolescence [38]. It is hypothesized that hypocortisolism may lead to neuroendocrine abnormalities and later health problems [52]. In a study of prenatal adversities on infants’ behavioral and cortisol reactivity during the first two days of life, the family’s household density was negatively associated with cortisol reactivity [51]. The direction of these findings are congruent with our finding that mother’s exposure to household density and poverty during pregnancy may dampen children’s sympathetic nervous system’s responses during the first five years of life. Children who come from low-socioeconomic childhood backgrounds, compared to high- socioeconomic backgrounds, may develop hypo-responsive ANS reactivity in response to repeated adversities [10] as an adaptive mechanism to reappraise stressors and subsequently reduce their risk of adult-onset cardiovascular disease [53]. Although the children in our study did not experience strong stability in their exposure to social support of socioeconomic adversities across the first five years of life, the prenatal adversities had a potent effect on their HR and sympathetic nervous system trajectories.

It is interesting in our study that prenatal social support adversity dampened offspring’s HR trajectory while socioeconomic adversity dampened offspring’s sympathetic nervous system. The children’s parasympathetic nervous system has been shown to be responsive to human social communication and engagement [54], [55], while the sympathetic nervous system has been shown to be responsive to socioeconomic adversity, such as living in poverty [29], [38]. The ANS influences HR such that a withdrawal of the parasympathetic nervous system and/or activation of the sympathetic nervous system increases HR. In this study, our findings support a stronger sympathetic influence on HR compared to the parasympathetic influence for children whose mothers experienced social support adversity. Our findings support other studies showing a relationship between socioeconomic adversity and dampened ANS reactivity [19].

In some studies, there is a curvilinear relationship between adversity and ANS reactivity where children exposed to the lowest and highest levels of adversity are similar but they differ from the children exposed to the mid-level of adversity [18], [56]. In this study, children with mothers who experienced one, not two, prenatal social support adversities had the steepest HR slope from 6 months to 5 years of age. The children with mothers who experienced one social support adversity had lower HR reactivity 6 months compared to children with mothers who experienced two social support adversities but by 5 years of age they had similar low HR reactivity. Although the children with mothers who experienced one social support adversity showed a significant change from 6 months to 5 years of age, the children with mothers who experienced no social support adversity showed the most plasticity in HR reactivity over time.

Studies of young children exposed to adversity show different effects on repeated HR or HR variability (i.e. a measure of the parasympathetic nervous system). One study supported our findings which showed there was no significant relationship between cumulative prenatal adversity at 16 weeks gestation and offspring’s resting RSA at 5 to 6 years of age [13]. On the other hand, a study of pregnant mothers who experienced hassles (e.g. not acute or major stressors) in mid-late pregnancy had fetuses’ at 24 to 38 weeks with high HR variability (i.e. parasympathetic nervous system) compared to fetuses whose mothers experienced fewer hassles [4]. In addition, a study of low-income pregnant African American women who experienced high prenatal stress had infants with lower neonatal HR variability than mothers experiencing less stress [12], [13]. Possibly, these inconsistent results show that the relationship between prenatal adversity and offspring’s parasympathetic activity may be age- (i.e. fetus, infant, 5–6 years) and/or context-dependent (i.e. hassles, cumulative stress).

In our study, the children whose mothers experienced no prenatal socioeconomic adversity were sympathetically more responsive to the challenges by 5 years of age compared to children whose mothers experienced any adversity. Children who are physiologically reactive living in low stress environments have sometimes been found to have the best physical and behavioral health outcomes [57]. The physiologically responsive children may be showing signs of adaptive engagement and sensitivity to their environment. These results support the need to explore the calibration of children’s ANS and HPA systems over time in cohort studies rather than cross-sectional studies [58]. In addition, future studies are needed to understand the mechanisms which can explain these findings and thus, we would need a larger sample so we can analyze both fixed and random effects and assess the changes in ANS and adversity at each age and over time. Possibly, postnatal parental behaviors, family environments and children’s exposure to repeated adversities may help explain the resilience and responsiveness shown for children whose mothers experienced no prenatal adversity.

There are different sensitive periods of fetal programming depending on the gestational age of the fetus at the time of the adversity. In a study of multiple stressors during pregnancy, high maternal cortisol during early pregnancy (e.g. 15 weeks) predicted accelerated infant mental development (i.e. steeper positive slope across first postnatal year) [59]. On the other hand, high maternal cortisol during late-pregnancy predicted elevated cortisol in infants after a painful heel-stick procedure within 24 hours of birth [6]. Thus, the sensitive periods during prenatal adversity may program fetuses differently depending on the outcome observed, such as mental and motor development versus HPA activity.

The children are also affected by the postnatal environment whereby differential human experiences systematically affect their health across their life, known as ‘biologic embedding’ [60], [61]. Although there was moderate stability in adversity experienced during the prenatal to postnatal periods, the relationship between the level of adversity and ANS cross-sectionally was not significant. It may be that the impact of adversity early in life has long lasting effects on the ANS over time, but not concurrently [62].

Although this study found novel results, there are several limitations. Since the research question separated the time between the mother’s prenatal adversity and child’s ANS development, we did not assess potential mediators or moderators of this relationship such as mother’s autonomic reactivity, maternal depression, maternal-child responsiveness, and repeated adversities. The ANS trajectories may be confounded by the different protocols or equipment and it is not possible to determine if the challenges were equally engaging and challenging at each age. The conditional likelihood/fixed effects model assumes a linear relationship between age and ANS reactivity measures yet this study only included four timepoints. We did not assess non-linear trajectories. The small number of repeated ANS measures restricted our model to utilize fixed slopes and intercepts which controlled for between-subject variability not within- subjects variability of the ANS trajectories. The conditional likelihood methods also restricted our ability to include potential confounders in the models, such as years lived in the U.S., family strengths, etc. These adversities were weakly or moderately correlated across the timepoints. Lastly, these findings may be generalizable only to similar populations of low-income Latino families living in agricultural communities.

The observed association between maternal adversity during pregnancy and ANS development represents the consequences of subtle adaptations in multiple organ systems to the intrauterine environment. The potential biological mechanisms underlying the developmental plasticity of the ANS include epigenetic processes and changes at the molecular, cellular, and organ level in the offspring [48]. In summary, these results are important and new in the field of ’prenatal programming’ since the timing of the prenatal adversities was separated from the later development of ANS responses in their children. This study explored important and understudied potential biologic pathways that may explain how prenatal adversity can ‘get under the skin’ of children to change their course of ANS development. Children’s biologic sensitivity to their environment [32], as measured by cardiac indices of the ANS in response to resting and challenging conditions, appear to be shaped early in life and exhibit plasticity under conditions of low adversity during the first five years of life.
